# A systematic review and meta-analysis of urinary biomarkers in myalgic encephalomyelitis/chronic fatigue syndrome (ME/CFS)

**DOI:** 10.1186/s12967-023-04295-0

**Published:** 2023-07-05

**Authors:** Asher Taccori, Rebekah Maksoud, Natalie Eaton-Fitch, Maharshi Patel, Sonya Marshall-Gradisnik

**Affiliations:** 1 School of Medicine, University of Notre Dame, Sydney, Australia; 2 National Centre for Neuroimmunology and Emerging Diseases (NCNED), Menzies Health Institute Queensland, Griffith University, Gold Coast, Australia; 3 Consortium Health International for Myalgic Encephalomyelitis, Griffith University, Gold Coast, Australia; 4 School of Pharmacy and Medical Science, Griffith University, Gold Coast, Australia; 5 School of Medicine and Dentistry, Griffith University, Gold Coast, Australia; 6 Menzies Health Institute Queensland, Gold Coast, Australia

## Abstract

**Background:**

Myalgic Encephalomyelitis/Chronic Fatigue Syndrome (ME/CFS) is a multifactorial illness that affects many body systems including the immune, nervous, endocrine, cardiovascular, and urinary systems. There is currently no universal diagnostic marker or targeted treatment for ME/CFS. Urine is a non-invasive sample that provides biomarkers that may have the potential to be used in a diagnostic capacity for ME/CFS. While there are several studies investigating urine-based biomarkers for ME/CFS, there are no published systematic reviews to summarise existing evidence of these markers. The aim of this systematic review was to compile and appraise literature on urinary-based biomarkers in ME/CFS patients compared with healthy controls.

**Methods:**

Three databases: Embase, PubMed, and Scopus were searched for articles pertaining to urinary biomarkers for ME/CFS compared with healthy controls published between December 1994 to December 2022. The final articles included in this review were determined through application of specific inclusion and exclusion criteria. Quality and bias was assessed using the Joanna Briggs Institute Critical Appraisal Checklist for Case Control Studies. A meta-analysis according to Cochrane guidelines was conducted on select studies, in particular, those that investigate urinary free cortisol levels in ME/CFS patients compared to healthy controls using the program STATA 17.

**Results:**

Twenty-one studies were included in this review. All of the studies investigated urinary-based markers in ME/CFS patients compared with healthy controls. The reported changes in urinary outputs include urinary free cortisol (38.10%), carnitine (28.6%), iodine (4.76%), and the metabolome (42.86%). In most cases, there was minimal overlap in the main outcomes measured across the studies, however, differences in urinary free cortisol between ME/CFS patients and healthy controls were commonly reported. Seven studies investigating urinary free cortisol were included in the meta-analysis. While there were significant differences found in urinary free cortisol levels in ME/CFS patients, there was also substantial heterogeneity across the included studies that makes drawing conclusions difficult.

**Conclusions:**

There is limited evidence suggesting a consistent and specific potential urinary-based biomarker for ME/CFS. Further investigations using more standardised methodologies and more stringent case criteria may be able to identify pathophysiological differences with diagnostic potential in ME/CFS patients compared with healthy controls.

**Supplementary Information:**

The online version contains supplementary material available at 10.1186/s12967-023-04295-0.

## Introduction

Myalgic Encephalomyelitis/Chronic Fatigue Syndrome (ME/CFS) is a chronic, multi-system illness characterised by a diverse range of symptoms such as post-exertional malaise (PEM) in combination with neurological, immunological, cardiovascular, and endocrine manifestations. This illness is estimated to impact 17–24 million people worldwide, approximately equating to 1% of the population [1]. Currently there is no laboratory-based diagnostic test and it is a condition determined on the basis of exclusion of any other potential medical explanation [2].

The most widely accepted case definitions currently in use to define and diagnose ME/CFS include Fukuda criteria (FC) (1994), Canadian Consensus Criteria (CCC) (2003), International Consensus Criteria (ICC) (2011). The Institute of Medicine Criteria (IOMC) is also sometimes utilised (2015) [3–6]. Autonomic manifestations including changes in urinary frequency and genitourinary disturbances are acknowledged in the CCC and ICC criteria, respectively [4, 5].

Many health-related characteristics can be observed through analysis of urine samples [7]. Urine also has the ability to optimise medical testing methods due to ease, accessibility, abundance, and convenience of collection [7]. Urine samples may also offer specific knowledge not assessed in blood samples relating to the involvement of the renal system, systemic waste products, urinary metabolites, or signs of local infection as commonly practised in the clinical setting in the form of dipsticks and microscopy urinalysis [8].

Research continues to seek biomarkers of this illness which may assist in a more consistent clinical diagnosis of ME/CFS [9]. Identifying consistent biomarkers may also assist in understanding the pathophysiology of ME/CFS and therefore offering insight into potential targeted treatment options [9]. Currently there is a significant body of research investigating blood-based markers; however, no systematic reviews have primarily focused on the biomarkers within the urine amongst ME/CFS patients [10].

This systematic review therefore aims to compile and appraise existing ME/CFS research on urinary biomarkers and to analyse this research to assess if any trends exist which may have implications relating to the pathogenesis or diagnosis of ME/CFS.

## Methods

### Literature search

This systematic literature review followed the process outlined in the “Preferred Reporting Items for Systematic Reviews and Meta-Analyses” (PRISMA) guidelines [11]. Only articles that specifically contained the keywords were included as recommended by Cochrane’s guidelines; therefore, no studies were handpicked. International Prospective Register of Systematic Reviews (PROSPERO, National Institute for Health Research) was used to screen published listings to assess originality of topic investigated. The protocol was prospectively registered on the server (ID: CRD42023389666) on the 16th of January 2023. Three search databases were utilised: PubMed, Embase and Scopus. These databases were systematically searched with the terms “Fatigue Syndrome, Chronic” in conjunction with urine, renal, filtrate, and urinalysis. Medical subject headings (MeSH) and full text terms were used to expand the search. Boolean operators ‘OR’ and ‘AND’ were applied to ensure all the required terms were included and to identify articles containing both an urinary search term and ME/CFS search terms. The full list of search terms are shown in Additional file 1. Initial independent searches were conducted by authors AT and RM using the same methodology on the 12th of December 2022. A subsequent and final search was conducted on 18th of January 2023.

### Inclusion and exclusion criteria

The systematic literature review included a search of all three databases with the following inclusion criteria: (1) original, full-text publications available in English (no reviews, duplicate studies, or case studies); (2) human observational studies conducted in adults (≥ 18 years old); (3) published following the introduction of the FC in 1994; (4) ME/CFS patients fulfil at least one of the following case definitions: FC (1994), CCC (2003), ICC (2011) or the IOMC (2015); (5) the studies investigated urinary products in ME/CFS compared with a HC group.

Articles were excluded if any of the following exclusion criteria applied: (1) articles that reported on non-original data including: reviews, duplicate studies or case studies or not written in the English language; (2) studies including interventional assessments that did not include baseline data (baseline data remained relevant to this literature review as it was recorded before the influence of an intervention); (3) studies involving participants that were under 18 years of age or non-human; (4) articles published before the establishment of the FC in December 1994 or that uses an alternative criteria other than FC, CCC, ICC, or IOMC; (5) studies that were outside the scope of this systematic literature review.

### Selection of studies

Any duplicate papers were deleted utilising the automated screening function via EndNote 20. A further two duplicate papers were deleted after manual screening. Once the initial screening of abstract and title was completed, full papers were reviewed to ensure they met the selection criteria. This process was undertaken independently by both authors AT and RM to confirm the final selection of papers was consistent between authors in order to reduce selection bias.

### Data extraction

The following data when available was extracted from each of the included publications: (1) author; (2) year; (3) study type; (4) ME/CFS diagnostic criteria; (5) sample size; (6) age of participants; (7) sex percentage; (8) body mass index (BMI); (9) illness duration; (10) urine collection method; (11) urinary product; (12) analysis method; and (13) study findings.

### Statistical analysis

A meta-analysis according to Cochrane guidelines was conducted on select studies using the program STATA 17. An inverse- variance, fixed- effect method approach was used. An I^2^ statistic was also calculated to measure heterogeneity between the studies. A fixed-effects model over a random-effects model was selected as there were only a small number of studies included in this meta-analysis and the outcome of interest: urinary free cortisol (UFC) was measured using similar methodologies across the studies, this suggests there may be a common effect. A sensitivity analysis was conducted to determine the influence of potential outliers.

### Quality assessment

The 2017 Joanna Briggs Institute (JBI) Critical Appraisal Checklist for Case Control Studies (CACCCS) checklist was used to assess the quality and bias of the reviewed papers. The JBI checklist items are as follows: (1) group matching, (2) source population, (3) criteria, (4) method of exposure, (5) assessment of exposure, (6)confounding variables identification, (7) mitigation of confounding variables, (8) measurement of outcomes, (9) exposure period selection, (10) statistical analysis. The JBI CACCCS quality assessment table and descriptions are compiled in Additional file 4.

Due to the exclusion of post intervention study data, JBI checklist numbers four, five and nine were not applicable. The remaining checklist items were assessed independently by both authors AT and RM and the results were compared and discussed to ensure consistency between both authors. As item 10 for two publications [12, 13] remained inconsistent following discussion between AT and RM, NEF also conducted a quality assessment for these items. The final quality analysis was deemed accordant by all authors.

## Results

**Fig. 1:**
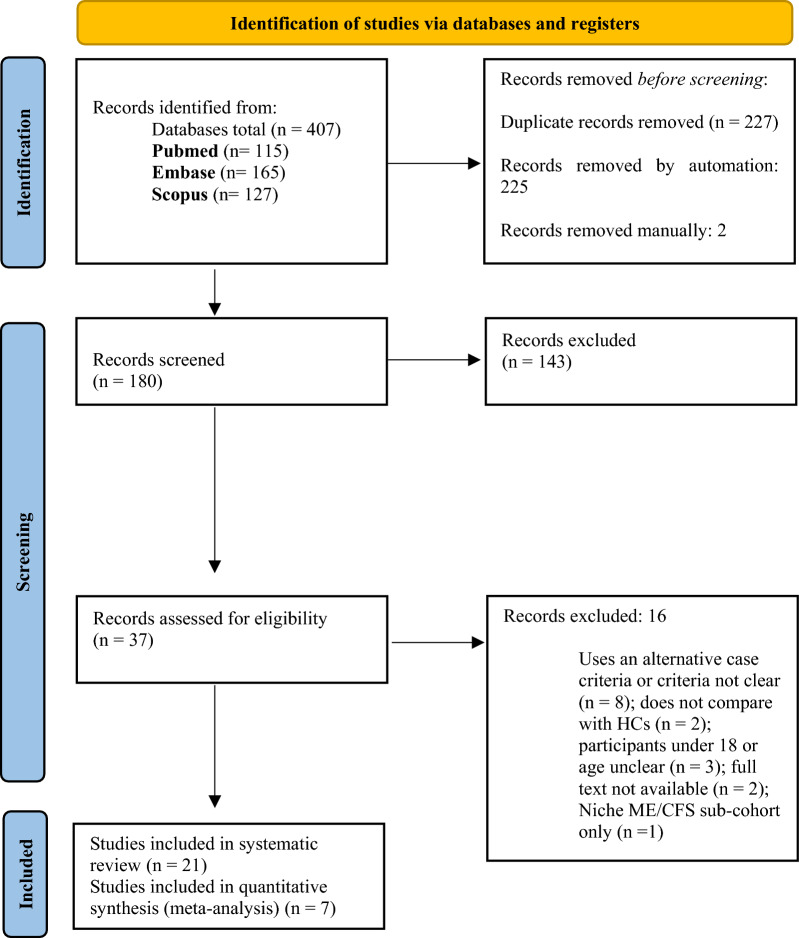
PRISMA flow diagram of literature search for included studies in this review of urinary product biomarkers in ME/CFS compared to HC

From the following databases: Pubmed (n = 115), Embase (n = 165), and Scopus (n = 127), a total of 407 publications were retrieved. Following duplicate removal and abstract and title screening the total number of publications included in this review was refined to 21 [12–32]. This search method was conducted according to the PRISMA guidelines, and the process has been outlined in Fig. 1. Seven studies were included in the meta-analysis [16, 17, 19, 20, 29–31]. All studies investigated UFC output in ME/CFS patients compared to HC [16, 17, 19, 20, 29–31].

### Overview of publications

The study and participant characteristics are summarised in Additional file 2. All the publications included in this review were observational case control studies that investigated urinary product abnormalities in ME/CFS compared with HC [12–32]. Two of the studies investigated potential therapeutics; however, only the baseline results prior to intervention were included in this review [17, 21]. The average number of ME/CFS patients and HC across the studies was 36.78 and 32.10, respectively. Only one of the studies reported on race where 93% of participants were Caucasian [25]. The average age of ME/CFS patients was 37.82 ± 9.19 and the average age of HC was 36.71 ± 7.54. Across the studies, 72.66% of ME/CFS patients and 67.31% of HC were female. Fourteen of the studies included patients that met a minimum of the FC for diagnosis [12, 16, 19–25, 28–32]. Four studies recruited patients diagnosed according to the CCC [14, 15, 26, 27], and three studies recruited patients diagnosed using the ICC [13, 17, 18]. No studies used the IOMC to diagnose patients. BMI and illness duration information was extracted from the publications and included in the results table; however, due to many studies not providing a value, an average was not calculated as the result may not be representative of all the included studies.

The timed long-term specimen collection method (24 h) was most prominently used to source urine whereby 14 studies used this method of sample collection [12, 13, 16–18, 20, 21, 25, 26, 28–32]. Four studies exclusively used a single first morning specimen urine collection method [14, 15, 24, 27]. Jones et al. collected urine samples using both a first morning and timed short-term (6 h) specimen collection method [22, 23]. Jerjes et al. used the timed short-term urine specimen collection method; however, this was repeated every 3 hours over a 15 h period [19]. Three studies reported no significant difference in average urine volumes between ME/CFS patients and HC [16, 18, 20]. McGregor et al. however, reported an association with higher urine volume and increased pain distribution in ME/CFS patients compared with HC [26].

### Literature reporting on changes in hormone or hormone substrates excreted from the urine

UFC was most frequently measured across all the studies included in the review. A total of eight studies investigated UFC in ME/CFS compared with HC (38.10%) [16, 17, 19–21, 25, 29, 30]. Most studies used a radioimmunoassay to measure cortisol levels [16, 17, 19, 20, 29, 30]. Inder et al. used enzyme-linked immunosorbent assay (ELISA) to measure urinary cortisol [31]. The method used to measure UFC was not described by Maloney et al. [25]. Four studies reported reduced UFC in ME/CFS patients compared with HC [16, 17, 20, 29]. Cleare et al. however, found that there were only significant differences in UFC in ME/CFS patients compared with HC in patients that reported not being on any medication [16]. Jerjes et al. measured cortisol and cortisone levels every 3 hours over a 15 h period [19]. The authors reported that there were significantly lower levels of both cortisol and cortisone at all timepoints except between 1800 and 2100 h in ME/CFS patients compared with HC [19]. Three studies reported no significant differences in UFC excretion between ME/CFS patients and HC [20, 30, 31]. Maloney et al. found that allostatic load was higher in ME/CFS patients compared with HC [25]. The authors identified that urinary cortisol was a key component of allostatic load that could accurately discriminate between ME/CFS patients and HC [25]. However, the level of UFC was not reported by Maloney et al. [25].

**Fig. 2:**
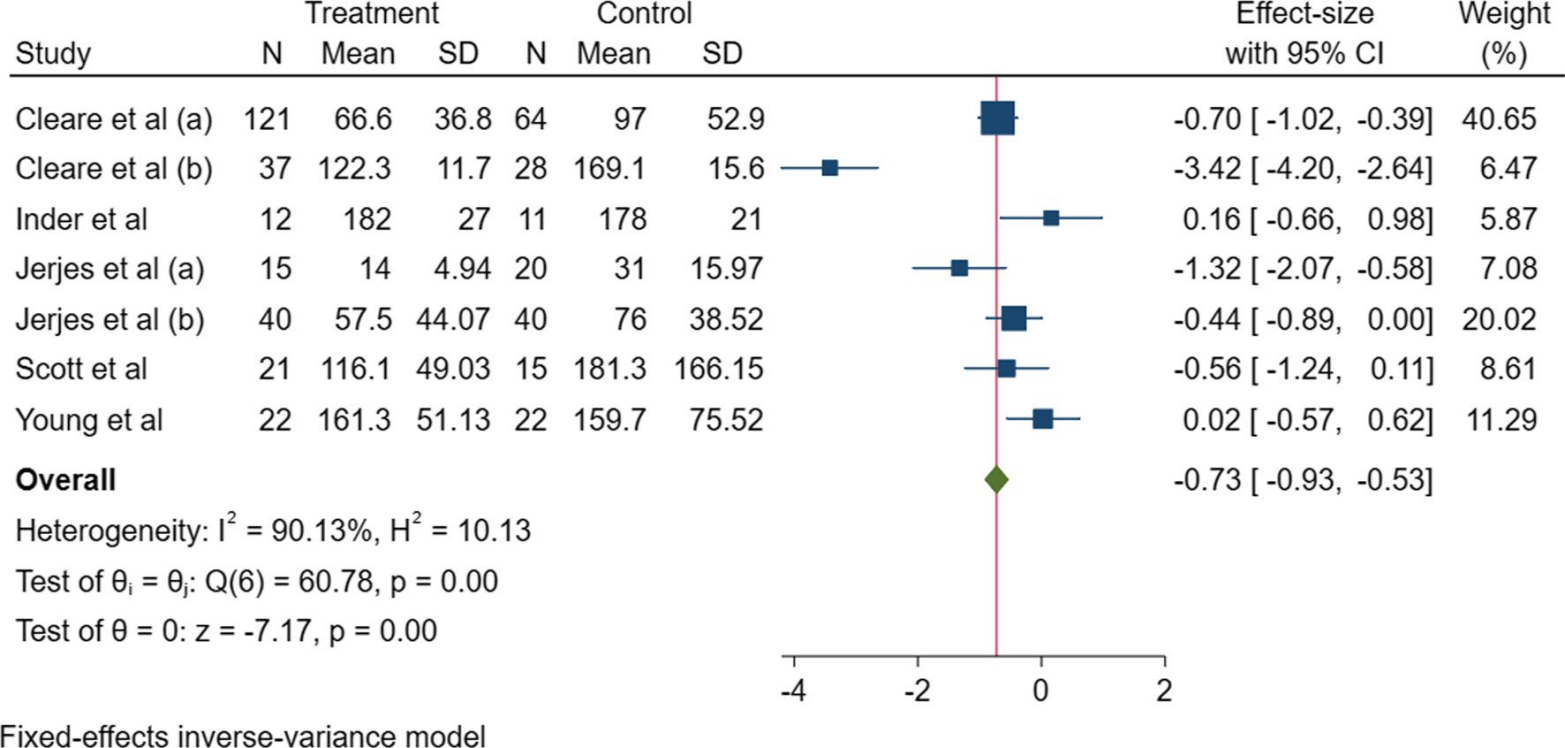
Fixed-effect inverse- variance model meta-analysis forest plot of studies investigating UFC in ME/CFS patients and HC. Significance is measured at a 95% confidence interval (p ≤ 0.05). z test, p- value = 0.00. Heterogeneity (I^2^) = 90.13% (test of θ_i_ shows p = 0.00). *CI* confidence interval, *H* heterogeneity, *ME/CFS* myalgic encephalomyelitis/chronic fatigue syndrome; *n* number, *SD* standard deviation, *UFC* urinary free cortisol

A meta-analysis was conducted on seven of the included studies that investigated UFC and findings were presented as a forest plot (Fig. 2) [16, 17, 19, 20, 29–31]. Maloney et al. was excluded from the analysis as they did not report a value for UFC [25]. A meta-analysis was only conducted on UFC as all other urinary measures did not measure the same outcomes. The z test found that there was a significant difference in UFC between ME/CFS and HC (p = 0.001), however, the level of heterogeneity between the studies was also at 90.13% and was significant (p = 0.001). A standard funnel plot representing small-study effects was also produced to illustrate potential deviations across the studies indicating potential publication bias (Additional file 3). Three studies: Cleare et al., Young et al., and Inder et al. effects deviate significantly from the estimated θ_iv_ [17, 30, 31].

Urinary cortisol metabolites were measured in three studies [20–22]. There were no significant differences found in urinary cortisol metabolite levels between ME/CFS patients and HC in any of the studies [19–21]. Cleare et al. measured 24 h urinary output of growth hormone between ME/CFS patients and HC. There was no significant difference in output between both groups [18]. Ruiz-Núñez et al. reported that 24 h urine iodine output was significantly lower in ME/CFS patients compared with HC [28].

### Literature reporting on changes in creatinine levels

Creatinine was also frequently investigated across the included studies in this review. Six studies investigated creatinine levels in ME/CFS patients compared with HC (28.6%) [12–15, 23, 24]. The technique used to measure creatinine varied between the studies. Two studies used the Jaffa method to measure participant urine creatinine levels [22, 24]. One study used capillary electrophoresis [12], another used a radio-enzymatic assay [23]. The methodology employed to measure creatinine levels by Lidbury et al. was unclear [13]. Two studies reported significant changes in creatinine excretion profile in ME/CFS patients compared with HC [12, 13]. Casado et al. compared creatinine concentrations in ME/CFS patients with and without comorbid fibromyalgia (FM) with HC. Urine creatinine concentration was significantly lower in ME/CFS/FM compared with HC [12]. Casado et al. also measured electrophoretic peaks representing creatinine and uric acid levels and found significant differences in patterns including peak amplitude and prevalence when comparing ME/CFS patients and HC [12]. Lidbury et al. found that 24 h urinary creatinine clearance was significantly lower in ME/CFS patients and in combination with serum urea and serum activin showed strong predictive capability (AUC: 0.963) in identifying ME/CFS patients from HC [13]. In contrast, Armstrong et al. found that creatinine levels were significantly higher in ME/CFS patients compared with HC [14, 15]. Maes et al. found associations between variance in urinary deoxyguanosine (8-OHdG) and urinary excretion of creatinine [24]. The levels of urinary creatinine in ME/CFS patients or HC; however, was not reported [24]. Two publications reported no significant differences in creatinine excretion profiles between both groups [22, 23]. Jones et al. found no significant differences in creatinine and free creatinine levels or total carnitine excretion rates in ME/CFS patients compared with HC [23]. Another study by Jones et al. also reported no significant differences in creatinine levels between ME/CFS patients and HC [22].

### Literature reporting on changes in urinary metabolites

A total of nine studies investigated urinary metabolites in ME/CFS patients compared with HC (42.86%) [14, 15, 19–22, 26, 27, 32]. Differences in levels of energy metabolism products in the urine were reported in three studies [14, 15, 27]. These three studies used nuclear magnetic resonance (NMR) spectroscopy to measure metabolites [14, 15, 27]. There was limited overlap with the metabolites measured. Significantly lower levels of pyruvate were described in one study [14] and lower urine acetate levels in ME/CFS patients compared with HC was described in two studies [14, 26]. McGregor et al. described significantly lower acetate levels in ME/CFS patients that experienced PEM in the seven days prior to urine collection or ME/CFS patients that did not [27]. Armstrong et al. conducted a correlation analysis between these urine metabolites and faecal/blood serum metabolites between ME/CFS and HC; however, significant associations were only observed in HC [15].

Changes in amino acid products in the urine are equivocal [14, 22, 32]. Two studies used gas chromatography to measure metabolites except Armstrong et al. who used NMR spectroscopy [14, 22, 32]. β-alanine was measured in two studies [22, 32]. Jones et al. reported significantly lower excreted β-alanine in ME/CFS patients compared with HC [22]. Hannestad et al. however, found no significant differences found in excretion levels of β-alanine between ME/CFS patients and HC [32]. Hannestad et al. also measured levels of γ-Aminobutyric acid (GABA) and reported no significance between ME/CFS patients and HC nor was there a difference in β-alanine excretion of GABA between the two groups [32]. Minimal overlap of the differences in amino acid metabolic products is reported in ME/CFS compared with HC, however, these are not significant between ME/CFS and HC. Significantly lower levels of the following amino acids were described in ME/CFS patients: hydroxyproline, histidine, valine, methionine, cystine, alanine, serine, and phenylalanine [14, 22].

In the three studies that investigated cortisol metabolites, no significant differences between ME/CFS patients and HC were found in any of the products [19–21].

### Quality analysis

Quality and bias were assessed using the CACCCS checklist. Items 4, 5, and 9 were not applicable as interventional studies were excluded from this review. All articles addressed item 6, including appropriately identifying confounding factors and item 8, that outcomes were assessed in a standard, valid, reliable way (100%) [12–32]. While every study identified confounding factors, only 19 of the studies (90.5%) offered strategies to mitigate these confounding factors [12–25, 28–32]. From the included studies, 14 (66.7%) appropriately matched ME/CFS patients and HC in particular regarding age- and sex [12–15, 17–23, 26–28, 30, 32]. Sixteen studies (76.2%) provided criteria used to identify ME/CFS patients and HC [12, 14–21, 24, 26–31] and 11 studies used appropriate statistical analysis (52.4%) [14, 15, 18, 19, 21–24, 26, 27, 31]. The least addressed criteria was checklist item two: where only nine (42.9%) of the included studies appropriately matched participants according to source population [16–21, 25, 30, 32].

## Discussion

This is the first study to systematically compile and appraise research for the potential application of urinary markers as diagnostic markers in ME/CFS.

This review included search terms also inclusive to urinary pathology; however, all included studies were focused on products excreted from the urine rather than structural changes in urinary-based mechanisms or onset of urinary pathological conditions. The search did not retrieve any publications on bacterial presence within the urine; however, urinary tract infections have been associated with ME/CFS and have been acknowledged in the ME/CFS case definitions including the CCC [4].

The average age of ME/CFS patients across the studies was 37.82 ± 9.19. The average age of onset of ME/CFS in literature is 33 [33]. The duration of illness across the studies ranged from 2.5 to 24.9 years. Due to such variability between the duration of illness, it is difficult to determine if the population age was representative of what is commonly reported in literature. Importantly, studies have shown that presentation of illness may be different depending on duration. The range in duration of the illness for studies was diverse, therefore this may have influenced the observed physiological profile of the urinary biomarkers for ME/CFS patients. Patients were not stratified based on duration of illness, hence this impact as a potential confounder remains unclear. Young et al. suggested that lack of basal activity differences observed in UFC between ME/CFS patients and HC may have been due to not including those who had ME/CFS for a long-term duration highlighting the potential role of illness duration on presentation of these markers [30].

The percentage of females recruited were higher compared with males for the studies included in this systematic review. This is supported by previous studies reporting females are more commonly diagnosed with ME/CFS [33]. Race was not frequently reported in these studies. Only one study provided information about the race of their participants and in this case, Caucasians made up 93% of the participants involved in the study [25].

The presence of comorbid conditions in ME/CFS is common. In this current review only two studies reported comorbid conditions or separated patients into subgroups according to their condition [12, 27]. Casado et al. compared in ME/CFS patients with and without fibromyalgia (FM) [12]. Independent studies have identified significant differences in UFC in fibromyalgia patients compared with HC. Hence, careful consideration determining comorbidities is paramount as a potential confounder, where these influences need to be accounted for or adjusted for [12]. McGregor et al. investigated metabolite levels in urine in ME/CFS patients who experienced or did not experience PEM seven days prior to urine collection [27]. A significant association with PEM was a lower serum level of the purine metabolite, hypoxanthine. Therefore, parameters measured may also be influenced by illness presentation and progression [27].

The most frequent case criteria used to define ME/CFS patients across the studies included in this systematic review was the FC [12, 16, 19–25, 28–32]. The FC is limited due to its broad and non-specific criteria and overlap with other clinical conditions [4, 5]. There is also significant heterogeneity shown between patients diagnosed using this definition that may reflect in the study findings making comparisons difficult [3]. Therefore, considerations for future experiments should involve use of more stringent case criteria used to select patients for recruitment, in particular the CCC and ICC case definition [4, 5].

Across the studies there were various methods used to source the urine from participants. Incorporation of different urinary collection methods can provide a diverse range of information. 24 h urine collections was the most prominent selection for urine collection [12, 13, 16–18, 20, 21, 25, 26, 28–32]. This long-term method for collection in particular over 24 h compared to 12 h is recognised as the gold standard, as it accounts for time lapse variability throughout the day. Mann et al. however, indicated that greater than 30% of collections are incomplete and do not represent the full 24 h excretion [34]. In all these cases there was no significant difference in urine volume identified as patients that did not have a complete collection were excluded.

The urinary-based marker, UFC was frequently reported in the studies evaluated [16, 17, 19–21, 25, 29, 30]. There was variability in results between some studies despite using similar methodologies. One study investigated UFC across several different time points throughout the day and found that UFC was consistently lower, although not significant, in ME/CFS patients at all time points except 1800–2100 h. Although UFC was measured over time, true longitudinal differences in UFC across a period of time (accounting for potential seasonal and other factors) has not been investigated in ME/CFS patients [19]. UFC is recognised as a primary biomarker for Cushing’s-related conditions, it is however, difficult to measure cortisol levels that influence intracellular receptors resulting in symptom presentation. Therefore, the use of UFC levels is difficult to ascertain as a reliable biomarker [35].

A meta-analysis was conducted to assess whether UFC can be used as a marker to effectively differentiate between ME/CFS patients and HC [16, 17, 19, 20, 29–31]. While the analysis found that there were significant differences in UFC in ME/CFS patients compared with HC, the heterogeneity across the studies was significant therefore the studies may not be comparable. Hence, the results need to be interpreted with caution [16, 17, 19, 20, 29–31]. Iodine was measured in one study, despite there being significant differences in levels of iodine measured through the urine in ME/CFS patients compared with HC. However, iodine is not produced within the body and is exclusively dependent on diet. Therefore, it is difficult to determine its specific influence or association with ME/CFS pathology [28].

Creatinine was also a key biomarker investigated across the included studies [12–15, 23, 24]. Both significantly lower and higher levels of creatinine were described in ME/CFS [12–15, 23, 24]. An increase in creatinine is associated with reduced kidney functioning, however, this marker cannot be used in isolation to determine kidney pathophysiology [36]. Low creatinine levels are associated with depleted muscle mass. While muscle loss is associated with bed rest [37] and 25% of ME/CFS patients are homebound or bedridden [38], reduced muscle mass is not yet a clinically established characteristic of ME/CFS and this association between creatinine excretion has not been investigated.

Huth et al. published a comprehensive systematic review on metabolomics in ME/CFS [10]. However, this previous review primarily focused on blood-based metabolites and also highlighted that even though metabolites measured throughout the urine can provide insight on levels of metabolites (in particular, deficiencies and excess in the blood that may impact homeostasis), further conclusive investigations focusing primarily on urinary biomarkers need to be undertaken as the results are equivocal [10]. Moreover, there were no standardised or universal methods used to measure urinary metabolites across the studies. The studies were difficult to compare due to differences in metabolite extraction and analysis method [10]. Excretion of products involved in energy processes may potentially indicate the involvement of disrupted energy metabolism in the pathomechanism of ME/CFS [14, 15, 27]. A systematic review by Holden et al. also investigated mitochondrial changes in ME/CFS; however, there are currently limited consistent findings that demonstrate the involvement of this network in ME/CFS pathology [39].

Outcomes such as health-related quality of life or symptom experience was reported in 12 studies [13, 16, 17, 19, 21–24, 26, 27, 31, 32]; however, only three studies conducted an association analysis determining the relationship between symptom presentation and/or experience and urinary outputs [13, 24, 26]. Therefore, it is difficult to determine whether these physiological observations are contributing to ME/CFS presentation; however, use of association studies may provide important insight and hence are a valuable consideration for inclusion in future studies.

Limitations across these studies include that these biomarkers are tertiary by-products therefore many markers that are investigated throughout the urine are non-specific and are presented in many other pathophysiological conditions. It is important to determine what upstream physiological pathways are impacted. All of these included studies were cross-sectional and did not represent long-term changes in these markers over time. These markers measured in the studies are also highly dynamic, and their levels are highly subjected to various external factors that are difficult to control for in a biological setting.

### Quality analysis

Quality varied across the studies. All studies assessed outcomes in a standard, valid and reliable way and identified potential confounding factors [12–32]. In many cases this was the presence of the The Diagnostic and Statistical Manual of Mental Disorders, Fifth Edition psychiatric condition, use of medications as well as the consumption of alcohol, and caffeine. Although all studies identified confounding variables, two studies did not provide methods for controlling for this source of variability [26, 27]. Shamim et al. found that there are sex specific differences in steroid metabolite excretion profiles [40]. Therefore, in order to make meaningful observations, sex- and age-matching is a necessary consideration. Menstruation also may influence the output of these urinary markers therefore some studies also further controlled for this potential source of variability. Eleven studies (52.4%) successfully addressed item 10 relating to statistical analysis [14, 15, 18, 19, 21–24, 26, 27, 31]. This was fulfilled through appropriate selection of test on the basis of data distribution through normality testing and adjustments for multiple comparisons. This item did not address whether a suitable sample size for statistical significance was determined as in many cases these studies are preliminary in nature and require validation in a larger sample. The least addressed item was reporting on whether patients and HC were matched according to source population; this is an important inclusion for future studies to ensure appropriately matched participants.

## Conclusions

This review highlights there is inconsistent evidence for the potential disruption of metabolic and hormonal processes as a consistent and specific potential biomarker for ME/CFS. These studies; however, only showcase a snapshot of data that can be extracted from urine. Many of the urinary biomarkers being measured are dynamic and are very susceptible to change through exposure to environmental or other non-disease related biological variables. These markers are also non-specific and are associated with other conditions, as they are a tertiary by-product it is difficult to determine particular disrupted upstream pathways associated with these urinary-based changes observed. Studies included in this review were also limited by sample size and lack of validation due to variable methodology. Further conduction of urine-based studies using alternative methodologies with more stringent recruitment criteria may provide more understanding on the pathophysiology of and diagnostic potential of urinary-based biomarkers in ME/CFS patients.

## Supplementary Information

**Additional file 1: **Raw search code.

**Additional file 2: **Participant, study characteristics, and primary findings.

**Additional file 3:** Meta-analysis small-study effects funnel plot of studies investigating UFC in ME/CFS patients and HC.

**Additional file 4: **The Joanna Briggs Institute Checklist for Case Control Studies table and descriptions.

## Data Availability

All data generated or analysed during this study are included in this published article [and its additional file information files].

## Abbreviations

BMI: Body mass index
CCC: Canadian consensus criteria
CACCCS: Critical appraisal checklist for case control studies
ELISA: Enzyme- linked immunosorbent assay
FM: Fibromyalgia
FC: Fukuda criteria
HC: Healthy controls
IOMC: Institute of medicine criteria
ICC: International consensus criteria
JBI: Joanna Briggs Institute
PROSPERO: International prospective register of systematic reviews
MeSH: Medical subject headings
ME/CFS: Myalgic encephalomyelitis/ chronic fatigue syndrome
PRISMA: Preferred reporting items for systematic reviews and meta-analyses
PEM: Post-exertional malaise
UFC: Urinary free cortisol

## References

[1] Lim EJ, Ahn YC, Jang ES, Lee SW, Lee SH, Son CG (2020). Systematic review and meta-analysis of the prevalence of chronic fatigue syndrome/myalgic encephalomyelitis (CFS/ME). J Transl Med.

[2] Deumer US, Varesi A, Floris V, Savioli G, Mantovani E, López-Carrasco P (2021). Myalgic Encephalomyelitis/chronic fatigue syndrome (ME/CFS): an overview. J Clin Med.

[3] Fukuda K, Straus SE, Hickie I, Sharpe MC, Dobbins JG, Komaroff A (1994). The chronic fatigue syndrome: a comprehensive approach to its definition and study international chronic fatigue syndrome study group. Ann Intern Med..

[4] Carruthers BM, Jain AK, De Meirleir KL, Peterson DL, Klimas NG, Lerner AM (2003). Myalgic Encephalomyelitis/Chronic Fatigue Syndrome. J Chronic Fatigue Syndrome.

[5] Carruthers BM, van de Sande MI, De Meirleir KL, Klimas NG, Broderick G, Mitchell T (2011). Myalgic encephalomyelitis: international consensus criteria. J Intern Med.

[6] Committee on the Diagnostic Criteria for Myalgic Encephalomyelitis/Chronic Fatigue S, Board on the Health of Select P, Institute of M. the national academies collection: reports funded by national institutes of health. beyond myalgic encephalomyelitis/chronic fatigue syndrome: redefining an Illness. Washington (DC): National Academies Press (US) Copyright 2015 by the National Academy of Sciences. All rights reserved. 2015.

[7] Jing J, Gao Y (2018). Urine biomarkers in the early stages of diseases: current status and perspective. Discov Med.

[8] Queremel Milani DA (2022). Urinalysis.

[9] Cortes Rivera M, Mastronardi C, Silva-Aldana CT, Arcos-Burgos M, Lidbury BA (2019). Myalgic encephalomyelitis/chronic fatigue syndrome: a comprehensive review. Diagnostics.

[10] Huth TK, Eaton-Fitch N, Staines D, Marshall-Gradisnik S (2020). A systematic review of metabolomic dysregulation in chronic fatigue syndrome/myalgic encephalomyelitis/systemic exertion intolerance disease (CFS/ME/SEID). J Transl Med.

[11] Page MJ, McKenzie JE, Bossuyt PM, Boutron I, Hoffmann TC, Mulrow CD (2021). The PRISMA 2020 statement: an updated guideline for reporting systematic reviews. BMJ.

[12] Casado B, Zanone C, Annovazzi L, Iadarola P, Whalen G, Baraniuk JN (2005). Urinary electrophoretic profiles from chronic fatigue syndrome and chronic fatigue syndrome/fibromyalgia patients: A pilot study for achieving their normalization. J Chromatogr, B: Anal Technol Biomed Life Sci.

[13] Lidbury BA, Kita B, Richardson AM, Lewis DP, Privitera E, Hayward S (2019). Rethinking ME/CFS diagnostic reference intervals via machine learning, and the utility of activin B for defining symptom severity. Diagnostics.

[14] Armstrong CW, McGregor NR, Lewis DP, Butt HL, Gooley PR (2015). Metabolic profiling reveals anomalous energy metabolism and oxidative stress pathways in chronic fatigue syndrome patients. Metabolomics.

[15] Armstrong CW, McGregor NR, Lewis DP, Butt HL, Gooley PR (2017). The association of fecal microbiota and fecal, blood serum and urine metabolites in myalgic encephalomyelitis/chronic fatigue syndrome. Metabolomics.

[16] Cleare AJ, Blair D, Chambers S, Wessely S (2001). Urinary free cortisol in chronic fatigue syndrome. Am J Psychiatry.

[17] Cleare AJ, Miell J, Heap E, Sookdeo S, Young L, Malhi GS (2001). Hypothalamo-pituitary-adrenal axis dysfunction in chronic fatigue syndrome, and the effects of low-dose hydrocortisone therapy. J Clin Endocrinol Metab.

[18] Cleare AJ, Sookdeo SS, Jones J, O'Keane V, Miell JP (2000). Integrity of the growth hormone/insulin-like growth factor system is maintained in patients with chronic fatigue syndrome. J Clin Endocrinol Metab.

[19] Jerjes WK, Peters TJ, Taylor NF, Wood PJ, Wessely S, Cleare AJ (2006). Diurnal excretion of urinary cortisol, cortisone, and cortisol metabolites in chronic fatigue syndrome. J Psychosom Res.

[20] Jerjes WK, Taylor NF, Peters TJ, Wessely S, Cleare AJ (2006). Urinary cortisol and cortisol metabolite excretion in chronic fatigue syndrome. Psychosom Med.

[21] Jerjes WK, Taylor NF, Wood PJ, Cleare AJ (2007). Enhanced feedback sensitivity to prednisolone in chronic fatigue syndrome. Psychoneuroendocrinology.

[22] Jones MG, Cooper E, Amjad S, Goodwin CS, Barron JL, Chalmers RA (2005). Urinary and plasma organic acids and amino acids in chronic fatigue syndrome. Clin Chim Acta.

[23] Jones MG, Goodwin CS, Amjad S, Chalmers RA (2005). Plasma and urinary carnitine and acylcarnitines in chronic fatigue syndrome. Clin Chim Acta.

[24] Maes M, Mihaylova I, Kubera M, Uytterhoeven M, Vrydags N, Bosmans E (2009). Increased 8-hydroxy-deoxyguanosine, a marker of oxidative damage to DNA, in major depression and myalgic encephalomyelitis / chronic fatigue syndrome. Neuroendocrinol Lett.

[25] Maloney EM, Gurbaxani BM, Jones JF, de Souza CL, Goertzel BN (2006). Chronic fatigue syndrome and high allostatic load. Pharmacogenomics.

[26] McGregor NR, Armstrong CW, Lewis DP, Butt HL, Gooley PR (2016). Widespread pain and altered renal function in ME/CFS patients. Fatigue Biomed Health Behav.

[27] McGregor NR, Armstrong CW, Lewis DP, Gooley PR (2019). Post-exertional malaise is associated with hypermetabolism, hypoacetylation and purine metabolism deregulation in ME/CFS cases. Diagnostics.

[28] Ruiz-Núñez B, Tarasse R, Vogelaar EF, Dijck-Brouwer DAJ, Muskiet FAJ (2018). Higher prevalence of "Low T3 Syndrome" in patients with chronic fatigue syndrome: a case-control study. Frontiers Endocrinol.

[29] Scott LV, Dinan TG (1998). Urinary free cortisol excretion in chronic fatigue syndrome, major depression and in healthy volunteers. J Affect Disord.

[30] Young AH, Sharpe M, Clements A, Dowling B, Hawton KE, Cowen PJ (1998). Basal activity of the hypothalamic-pituitary-adrenal axis in patients with the chronic fatigue syndrome (Neurasthenia). Biol Psychiat.

[31] Inder WJ, Prickett TC, Mulder RT (2005). Normal opioid tone and hypothalamic-pituitary-adrenal axis function in chronic fatigue syndrome despite marked functional impairment. Clin Endocrinol (Oxf).

[32] Hannestad U, Theodorsson E, Evengård B (2007). β-Alanine and γ-aminobutyric acid in chronic fatigue syndrome. Clin Chim Acta.

[33] Bakken IJ, Tveito K, Gunnes N, Ghaderi S, Stoltenberg C, Trogstad L (2014). Two age peaks in the incidence of chronic fatigue syndrome/myalgic encephalomyelitis: a population-based registry study from Norway 2008–2012. BMC Med.

[34] Mann SJ, Gerber LM (2019). Addressing the problem of inaccuracy of measured 24-hour urine collections due to incomplete collection. J Clin Hypertens (Greenwich).

[35] Fleseriu M, Bancos I, Katz D (2021). A double-blind, randomized, placebo-controlled trial of SPI-62 safety and efficacy for the treatment of Cushing’s syndrome. Endocrine Abstr.

[36] Gounden VBH, Jialal I (2022). Renal function tests.

[37] English KL, Paddon-Jones D (2010). Protecting muscle mass and function in older adults during bed rest. Curr Opin Clin Nutr Metab Care.

[38] Chu L, Valencia IJ, Garvert DW, Montoya JG (2019). Onset patterns and course of myalgic encephalomyelitis/chronic fatigue syndrome. Front Pediatr.

[39] Holden S, Maksoud R, Eaton-Fitch N, Cabanas H, Staines D, Marshall-Gradisnik S (2020). A systematic review of mitochondrial abnormalities in myalgic encephalomyelitis/chronic fatigue syndrome/systemic exertion intolerance disease. J Transl Med.

[40] Shamim W, Yousufuddin M, Bakhai A, Coats AJ, Honour JW (2000). Gender differences in the urinary excretion rates of cortisol and androgen metabolites. Ann Clin Biochem.

