# Supplementary material for: A systematic review and meta-analysis of urinary biomarkers in myalgic encephalomyelitis/chronic fatigue syndrome (ME/CFS)
Source: J Transl Med. 2023 Jul 5;21:440. doi: 10.1186/s12967-023-04295-0 (PMC10320942; doi:10.1186/s12967-023-04295-0)
Supplement: Supplementary file 1 — Additional file 1: Raw search code. [file 12967_2023_4295_MOESM1_ESM.docx]

**Additional file 1.** raw search code

| **Database** | **Code** |
| --- | --- |
| PubMed | (("Chronic Fatigue Syndrome"[title/abstract] OR "Myalgic Encephalomyelitis"[title/abstract] OR “Encephalomyelitis, Myalgic”[title/abstract] OR “Chronic Fatigue Syndromes”[title/abstract] OR “Fatigue Syndromes, Chronic”[title/abstract] OR “Chronic Fatigue-Fibromyalgia Syndrome”[title/abstract] OR “Chronic Fatigue Fibromyalgia Syndrome”[title/abstract] OR “Chronic Fatigue Fibromyalgia Syndromes”[title/abstract] OR “Fatigue-Fibromyalgia Syndrome, Chronic”[title/abstract] OR “Fatigue-Fibromyalgia Syndromes, Chronic”[title/abstract] OR “Postviral Fatigue Syndrome”[title/abstract] OR “Infectious Mononucleosis-Like Syndrome, Chronic”[title/abstract] OR “Infectious Mononucleosis Like Syndrome, Chronic”[title/abstract] OR “Royal Free Disease”[title/abstract] OR “Chronic Fatigue and Immune Dysfunction Syndrome”[title/abstract] OR “Chronic Fatigue Disorder”[title/abstract] OR “Chronic Fatigue Disorders”[title/abstract] OR “Fatigue Disorder, Chronic”[title/abstract] OR “Fatigue Disorders, Chronic”[title/abstract] OR "systemic exertion intolerance"[title/abstract] OR “Fatigue Syndrome, Postviral”[title/abstract] OR “Fatigue Syndromes, Postviral”[title/abstract] OR “Postviral Fatigue Syndromes”[title/abstract])) AND ((urinary[Title/Abstract] OR urine[Title/Abstract] OR urinalysis[Title/Abstract] OR urinat* [Title/Abstract] OR urinalyses [Title/Abstract] OR urin*[Title/Abstract] OR mictur*[Title/Abstract] OR renal[Title/Abstract] OR filtrate[Title/Abstract])) |
| Scopus | ((TITLE-ABS("Chronic Fatigue Syndrome") OR TITLE-ABS("Myalgic Encephalomyelitis") OR TITLE-ABS(“Encephalomyelitis, Myalgic”) OR TITLE-ABS(“Chronic Fatigue Syndromes”) OR TITLE-ABS(“Fatigue Syndromes, Chronic”) OR TITLE-ABS(“Chronic Fatigue-Fibromyalgia Syndrome”) OR TITLE-ABS(“Chronic Fatigue Fibromyalgia Syndrome”) OR TITLE-ABS(“Chronic Fatigue Fibromyalgia Syndromes”) OR TITLE-ABS(“Fatigue-Fibromyalgia Syndrome, Chronic”) OR TITLE-ABS(“Fatigue-Fibromyalgia Syndromes, Chronic”) OR TITLE-ABS(“Postviral Fatigue Syndrome”) OR TITLE-ABS(“Infectious Mononucleosis-Like Syndrome, Chronic”) OR TITLE-ABS(“Infectious Mononucleosis Like Syndrome, Chronic”) OR TITLE-ABS(“Royal Free Disease”) OR TITLE-ABS(“Chronic Fatigue and Immune Dysfunction Syndrome”) OR TITLE-ABS(“Chronic Fatigue Disorder”) OR TITLE-ABS(“Chronic Fatigue Disorders”) OR TITLE-ABS(“Fatigue Disorder, Chronic”) OR TITLE-ABS(“Fatigue Disorders, Chronic”) OR TITLE-ABS("systemic exertion intolerance") OR TITLE-ABS(“Fatigue Syndrome, Postviral”) OR TITLE-ABS(“Fatigue Syndromes, Postviral”) OR TITLE-ABS(“Postviral Fatigue Syndromes”))) AND ((TITLE-ABS(“urinary”) OR TITLE-ABS(“urine”) OR TITLE-ABS(“urinalysis”) OR TITLE-ABS(“urinat*”) OR TITLE-ABS(“urinalyses”) OR TITLE-ABS(“urin*”) OR TITLE-ABS(“mictur*”) OR TITLE-ABS(“renal”) OR TITLE-ABS(“filtrate”))) |
| **Embase** | (("Chronic Fatigue Syndrome":ab,ti OR "Myalgic Encephalomyelitis":ab,ti OR “Encephalomyelitis, Myalgic”:ab,ti OR “Chronic Fatigue Syndromes”:ab,ti OR “Fatigue Syndromes, Chronic”:ab,ti OR “Chronic Fatigue-Fibromyalgia Syndrome”:ab,ti OR “Chronic Fatigue Fibromyalgia Syndrome”:ab,ti OR “Chronic Fatigue Fibromyalgia Syndromes”:ab,ti OR “Fatigue-Fibromyalgia Syndrome, Chronic”:ab,ti OR “Fatigue-Fibromyalgia Syndromes, Chronic”:ab,ti OR “Postviral Fatigue Syndrome”:ab,ti OR “Infectious Mononucleosis-Like Syndrome, Chronic”:ab,ti OR “Infectious Mononucleosis Like Syndrome, Chronic”:ab,ti OR “Royal Free Disease”:ab,ti OR “Chronic Fatigue and Immune Dysfunction Syndrome”:ab,ti OR “Chronic Fatigue Disorder”:ab,ti OR “Chronic Fatigue Disorders”:ab,ti OR “Fatigue Disorder, Chronic”:ab,ti OR “Fatigue Disorders, Chronic”:ab,ti OR "systemic exertion intolerance":ab,ti OR “Fatigue Syndrome, Postviral”:ab,ti OR “Fatigue Syndromes, Postviral”:ab,ti OR “Postviral Fatigue Syndromes”:ab,ti)) AND ((urinary:ab,ti OR urine:ab,ti OR urinalysis:ab,ti OR urinat*:ab,ti OR urinalyses:ab,ti OR urin*:ab,ti OR mictur*:ab,ti OR renal:ab,ti OR filtrate:ab,ti)) |
