# Supplementary material for: A systematic review and meta-analysis of urinary biomarkers in myalgic encephalomyelitis/chronic fatigue syndrome (ME/CFS)
Source: J Transl Med. 2023 Jul 5;21:440. doi: 10.1186/s12967-023-04295-0 (PMC10320942; doi:10.1186/s12967-023-04295-0)
Supplement: Supplementary file 2 — Additional file 2: Participant, study characteristics, and primary findings. [file 12967_2023_4295_MOESM2_ESM.docx]

**Additional file 2.** Participant, study characteristics, and primary findings

| **Author** | **Study type** | **Criteria** | **Sample size** |  | **Age (years, mean ± SD)** |  | **Sex (female %)** |  | **BMI (kg/m^2^) (mean ± SD)** |  | **Illness duration (years)** | **Urine collection method** | **Urinary product** | **Analysis method** | **Findings** |
| --- | --- | --- | --- | --- | --- | --- | --- | --- | --- | --- | --- | --- | --- | --- | --- |
|  |  |  | **ME/CFS** | **HC** | **ME/CFS** | **HC** | **ME/CFS** | **HC** | **ME/CFS** | **HC** | **ME/CFS** |  |  |  |  |
| Armstrong et al. (2015) (14) | Observational Case Control | CCC | 34 | 25 | 34.9 ± 1.8 SE | (33.0 ± 1.6 SE | 100.0% | 100.0% | 24.0 ± 0.81 SE | 23.0 ± 0.74 SE | NR | First morning specimen | Urinary metabolites | Nuclear magnetic resonance (NMR) spectroscopy | Thirty metabolites were identified and quantified in the urine. The absolute concentrations of five urinary metabolites significantly lower in ME/CFS and HC: acetate (p <0.003), alanine (p = 0.049), formate (p = 0.002), pyruvate (p = 0.034), and serine (p = 0.034). Eight metabolites were significantly altered within the relative abundance dataset for urine. acetate (p = 0.025) alanine (p = 0.008),formate (p = 0.026), pyruvate (p = 0.001), serine (p =0.008), and valine (p = 0.026) were lower in ME/CFS patients compared to HC while allantoin (p = 0.011) and creatinine (p = 0.025) were higher in ME/CFS patients. |
| Armstrong et al. (2017) (15) | Observational Case Control | CCC | 34 | 25 | 34.9 ± 1.8 SE | 33.0 ± 1.6 SE | 100.0% | 100.0% | 24.0 ± 0.81 SE | 23.0 ± 0.74 SE | NR | First morning specimen | Urinary metabolites | Nuclear magnetic resonance (NMR) spectroscopy | Fecal short chain fatty acids negatively correlated with serum and urine gluconeogenesis substrates in HC (-0.4 >r), however there was no correlation in ME/CFS patients (-0.4 < r < 0.4). Other Streptococcus species negatively correlated with urine amino acids, however, only in HC (-0.4 > r). |
| Casado et al. (2005) (12) | Observational Case Control | FC | 11 | 20 | NR | NR | 55.0% | 50.0% | NR | NR | NR | Timed long-term specimen (24-hr) | Urinary creatinine concentration | Capillary electrophoresis | Urine creatinine concentration was significantly lower in ME/CFS/ FM (0.78 mg/mL) compared to ME/CFS (1.62mg/mL) and HC (1.23 mg/mL, p < 0.001) at mAU214 nm.  Electrophoretic peaks representing creatinine and uric acid were compared between ME/CFS and HC. Peak 21 was significantly lower in ME/CFS and ME/CFS/FM groups compared to HC (p = 0.027). Peak 1 was significantly higher in ME/CFS/FM and Peak 24 was lower in ME/CFS patients compared to HC (p = 0.031).  The following peaks: 12 and 39 were more prevalent in ME/CFS than HC, however, peak 34 was less prevalent in ME/CFS patients. The following peaks were more prevalent in ME/CFS compared to HC: 3, 6, 13, 21, 24, 27, and 41 (no p- value). |
| Cleare et al. (2000) (18) | Observational Case Control | ICC | 37 | 37 | 33.8 ± 12 | 30.8 ± 6.4 | 67.57 | 67.57% | 23.8 ± 3.8 | 23.8 ± 3.1 | 42 ± 2.92 | Timed long-term specimen (24-hr) | 24-hr urinary GH excretion (pg/24 h) | two-site immunoradiometric assay | There were no significant differences in 24-hour urinary output of growth hormone in ME/CFS patients (62.9 pg/ 24 h) compared to HC (74.8 pg/24 h). The mean collected volume of urine over 24 hours also did not differ between the two groups |
| Cleare et al. (2001a) (16) | Observational Case Control | FC | 121 | 64 | 39.5 ± 11.2 | 33.9 ± 9.0 | 64 | 56.0% | NR | NR | 5.4 ± 4.3 | Timed long-term specimen (24-hr) | UFC | Technicon Immuno-1 assay (radioimmunoassay) | ME/CFS patients that were medication free had significantly lower UFC compared to HC (p < 0.0005). There were no significant differences between mean urinary volumes. |
| Cleare et al. (2001b) (17) | Observational Case Control | ICC | 37 | 28 | Mean: 33.8 (95% CI 30.3–37.3) | Mean: 32.4 (95% CI 28.5–36.3) | 67.57% | 50.0% | Mean: 23.3 (95% CI 22.1–24.2) | Mean: 24.4 (95% CI 23.0–25.8) | <8.3 years (8.33) | Timed long-term specimen (24-hr) | UFC | Radioimmunoassay | ME/CFS patients (122.3 ± 11.7 nmol/d) had significantly lower UFC values (p = 0.025) compared to HC (169.1 ± 15.6 nmol/d), respectively (F = 5.73, df (1, 23), P = 0.025).” |
| Hannestad et al. (2007) (32) | Observational Case Control | FC | 28 | 43 | Mean Females: 39.6 (range 25.5-53.5)  Mean Males: 40.4 (range 30.1-53.3) | Females: Mean: 41.9 (range 28.9-54.1)  Males: Mean: 40.6 (range 32.2-48.3) | 71.43% | 55.81% | NR | NR | Mean: 7.4 (range 1.5 to  25) | Timed long-term specimen (24-hr) | 24hr urine excretion of GABA and β-alanine | Isotope dilution gas chromatography mass spectrometry | There were no significant differences found in excretion of β-alanine or GABA between ME/CFS patients and HC male and female subgroups. There was no significant difference in β-alanine excretion of GABA in ME/CFS patients compared to HC. |
| Inder et al. (2005) (31) | Observational Case Control | FC | 12 | 11 | NR | NR | NR | NR | NR | NR | NR | Timed long-term specimen (24-hr) | UFC | ELISA | There were no significant differences in basal levels of 24hr UFC in ME/CFS patients (182 ± 27nmol/ 24 hr) compared to HC (178 ± 21 nmol/24 hr). |
| Jerjes et al. (2006a) (19) | Observational Case Control | FC | 15 | 20 | 35 ± 7.9 | 33 ± 11.3 | 53.33% | 50.0% | 24.4 ± 50 | 24.2 ± 4.6 | 2.7 ± 0.6 | Timed short-term specimen (every 3-hr over 15 hour period) | cortisol and cortisone levels | Radioimmunoassay | There was significantly lower cortisol and cortisone in ME/CFS patients compared to HC at all time points except 1800-2100 h. There was no significant overall difference in the ratio of UFC and cortisone throughout the day between ME/CFS and HC. There was no significant differences in ratio of urinary metabolites assessed. |
| Jerjes et al. (2006b) (20) | Observational Case Control | FC | 40 | 40 | 34.0 ± 1.6 | 32.6 ± 1.5 | 50.0% | 50.0% | 23.6 ± 0.7 | 23.7 ± 0.7 | 2.1 ± 0.1 | Timed long-term specimen (24-hr) | UFC  Cortisol metabolites | Radioimmunoassay and high- resolution gas chromatography | There were no significant differences in total cortisol metabolite (TCM) or cortisol metabolite ratios between ME/CFS patients and HC. There were no significant differences in UFC levels across all groups. |
| Jerjes et al. (2007) (21) | Observational Case Control | FC | 15 | 20 | 35.0 ± 7.9 | 32.0 ± 11.4 | 53.30% | 50.0% | 24.4 ± 5.0 | 23.7 ± 4.3 | 2.7 ± 0.6 | Timed long-term specimen (24-hr) | UFC  Cortisol metabolites | high- resolution gas chromatography | There were no significant differences found in urinary cortisol metabolites between ME/CFS and HC. |
| Jones et al. (2005a) (23) | Observational Case Control | FC | 31 | 31 | Male: 42 (26-63)  Female: 42 21-84) | 46 (20-66)  42 (26-79) | 61.29% | 61.29% | NR | NR | NR | First morning and timed short-term specimen (6-hr) (two collections) | creatine or carnitine levels | radio-enzynamic assay | There was no significant difference in creatinine or free carnitine levels or total carnitine excretion rates in ME/CFS patients compared to HC. |
| Jones et al. (2005b) (22) | Observational Case Control | FC | 30 | 30 | Male: 44 (26-63)  Female: 45 (26-84) | Male: 45 (20-66)  Female: 45 (26-79) | 63.33% | 63.33% | NR | NR | NR | First morning and timed short-term specimen (6-hr) (two collections) | Urinary Metabolites  Creatinine | Jaffe method  programmed temperature capillary gas chromatography | ME/CFS patients had significantly lower levels of the following amino acids compared to HC: β-alanine (p < 0.05), hydroxyproline (p < 0.001), histidine (p = 0.05), methionine (p < 0.01), cystine (p < 0.01), phenylalanine (p < 0.01). There were no significant differences in creatinine levels for various organic acids. |
| Lidbury et al. (2019) (13) | Observational Case Control | ICC | 80 | 17 | 39.3-56.0 | 29.0-51.0 | NR | NR | NR | NR | NR | Timed long-term specimen (24-hr) | 24-hour urinary creatinine | NR | 24-hour urinary creatinine clearance was significantly lower in ME/CFS patients (p ≤ 0.05) and in combination with serum urea, and serum activin showed strong predictive capability of detecting ME/CFS patients compared to HC (AUC: 0.963). |
| Maes et al. (2009) (24) | Observational Case Control | FC | 44 | 17 | 36.6 ± 14.2 | 45.1 ± 9.2 | 95.45% | 70.59% | NR | NR | NR | First morning specimen | urinary excretion of deoxyguanosine (8-OHdG) and creatinine | kinetic colorimetric assay - Jaffe method  ELISA | 49.0% of the variance in urinary deoxyguanosine (8-OHdG) was explained by urinary excretion of creatinine using regression analysis (r= 0.71, p < 0.005). There was an association found between 8-OHdG and scores of the FibroFatigue scale: sadness (p = 0.04) and experience of infection (p = 0.03). |
| Maloney et al. (2006) (25) | Observational Case Control | FC | 43 | 60 | 50.6 ± 8.7 | 50.5 ± 8.6 | 83.7% | 80.0% | 39.4 ± 4.4 | 28.6 ± 4.9 | NR | Timed long-term specimen (24-hr) | 24-hour urinary cortisol | NR | ME/CFS patients were more likely to have a higher allostatic load index compared to HC. This association increased in a linear trend (p = 0.06). the components of allostatic load that best discriminate against ME/CFS patients from HC include: waist: hip ratio, aldosterone and urinary cortisol (no p-value provided) |
| McGregor et al. (2016) (26) | Observational Case Control | CCC | Sub-populations of ME/CFS patients:  Facial pain: 22  No facial pain: 25 | 25 | Facial pain: 39.5 (15.8) *  No facial pain: 39.5 (15.6) | 33.6 (7.8) | Facial pain: 81.80%  No facial pain: 87.50% | 96.0% | Facial pain: 23.0 ± 3.4  No facial pain: 25.4 ± 6.8 | 23.1 ± 2.6 | Facial pain: 11.1 (7.3)  No facial pain: 11.8 (10.4) | Timed long-term specimen (24-hr) | Urinary Metabolites | NMR spectroscopy | Lower serum essential amino acids, urea, serum sodium and higher serum glucose and 24- hour urine volume was associated with an increase in pain distribution (r-value: not recorded, p < 0.01). |
| McGregor et al. (2019) (27) | Observational Case Control | CCC | Sub-populations of ME/CFS patients:  No PEM: 11  PEM: 35 | 25 | No PEM: 30.9 ± 9.6  PEM: 42.1 ± 16.3 | 33.6 ± 7.8 | No PEM: 100.0%  PEM: 80.0% | 96.0% | No PEM: 100.0%  PEM: 80.0% | 23.1 ± 2.6 | No PEM: 22.7 ± 3.6  PEM: 24.9 ± 6.1 | First morning specimen | Urinary metabolites | NMR spectroscopy | In urine, ME/CFS patients had significantly lower acetate compared to HC in both PEM (2.5-fold lower) and non-PEM (1.5- fold lower) groups. Serum hypoxanthine: urate ratio was lower in both PEM (3.5-fold lower) and non-PEM (5.4-fold lower) ME/CFS groups. The hypoxanthine: urate ratio was positively correlated with serum lactate (r = 0.77, p < 0.001) , the purine ring precursor amino acids (r = 0.54, p < 0.001), acetate (r = 0.49, p < 0.001), and total serum amino acids (r = 0.38, p < 0.006) as well as negatively correlated with serum glucose (r = -0.48, p < 0.001) |
| Ruiz-Núñez et al. (2018) (28) | Observational Case Control | / FC | 98 | 99 | 43 (21-69)* | 39 (19-65)* | 78.57% | 76.77% | 22 (18-34)* | 23 (18-33)* | NR | Timed long-term specimen (24-hr) | urine iodine | Inductively Coupled Plasma Mass Spectrometry (ICP-MS 7700x) | 24-hr urine iodine output was also significantly lower in ME/CFS patients compared to HC (p < 0.001). |
| Scott et al. (1998) (29) | Observational Case Control | FC | 21 | 15 | 36.1 ± 2.9 | 33.4 ± 2.2 | 66.67% | 60.0% | NR | NR | NR | Timed long-term specimen (24-hr) | UFC | Radioimmunoassay | UFC excretion was significantly lower in ME/CFS patients compared to HC (p < 0.01) |
| Young et al. (1998) (30) | Observational Case Control | FC | 22 | 22 | 39 ± 8.8 | 38 ± 8.0 | 45.45% | 45.45% | NR | NR | 2.5 ± 1.25 | Timed long-term specimen (24-hr) | UFC | Radioimmunoassay | There were no significant differences in 24-hour UFC excretion between ME/CFS patients and HC. |

*median

*Abbreviations: BMI, Body mass index; CCC, Canadian Consensus Criteria; CI, Confidence interval; 8-OHdG Deoxyguanosine; ELISA, Enzyme-linked immunosorbent assay; FM, Fibromyalgia; FC, Fukuda criteria; GABA, Gamma-aminobutyric acid; GH, Growth hormone; HC, Healthy control; ICC, International Consensus Criteria; ME/CFS, Myalgic Encephalomyelitis/ Chronic Fatigue Syndrome; NR, Not recorded; NMR, Nuclear magnetic resonance; PEM, Post-exertional malaise; SE, Standard error; UFC, Urinary free cortisol*
