# Supplementary figures and images for: A systematic review and meta-analysis of urinary biomarkers in myalgic encephalomyelitis/chronic fatigue syndrome (ME/CFS)
Source: J Transl Med. 2023 Jul 5;21:440. doi: 10.1186/s12967-023-04295-0 (PMC10320942; doi:10.1186/s12967-023-04295-0)

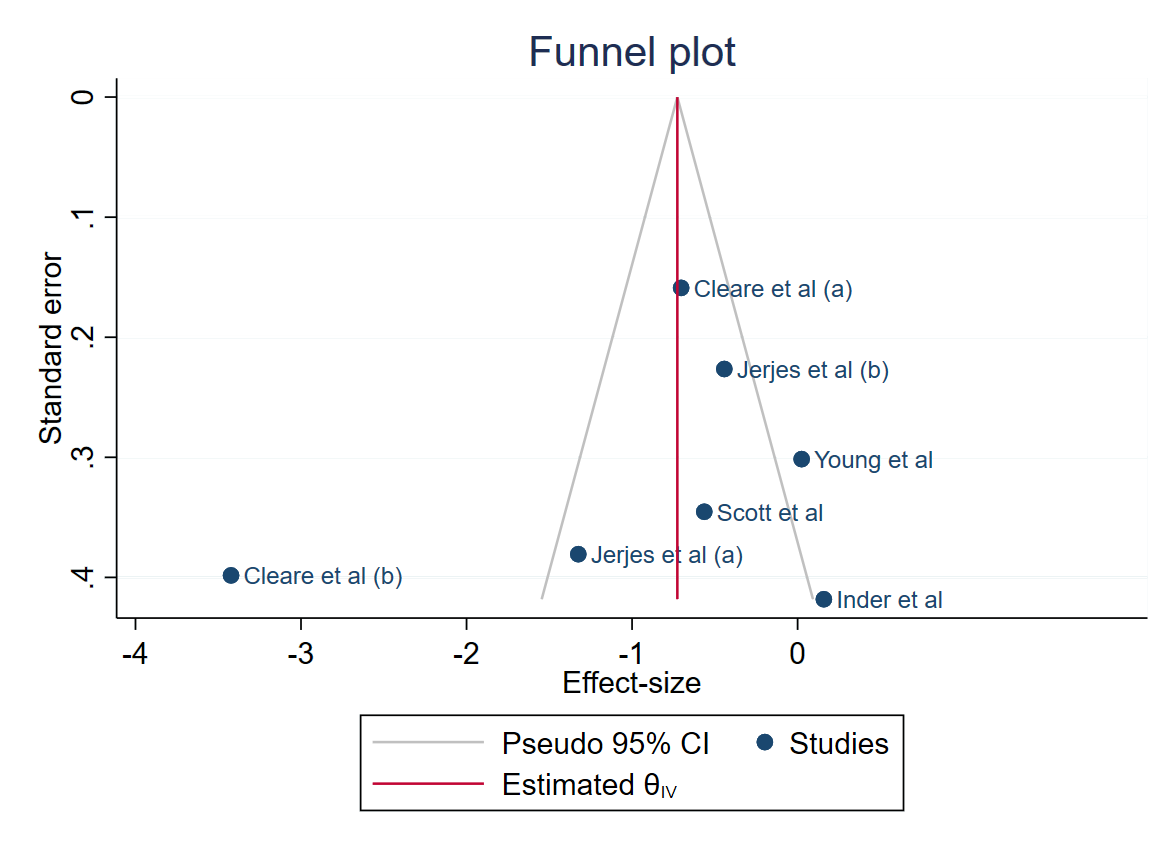

Supplement: Supplementary file 3 — Additional file 3: Meta-analysis small-study effects funnel plot of studies investigating UFC in ME/CFS patients and HC. [file 12967_2023_4295_MOESM3_ESM.docx]
