# Supplementary material for: A systematic review and meta-analysis of urinary biomarkers in myalgic encephalomyelitis/chronic fatigue syndrome (ME/CFS)
Source: J Transl Med. 2023 Jul 5;21:440. doi: 10.1186/s12967-023-04295-0 (PMC10320942; doi:10.1186/s12967-023-04295-0)
Supplement: Supplementary file 4 — Additional file 4: The Joanna Briggs Institute Checklist for Case Control Studies table and descriptions. [file 12967_2023_4295_MOESM4_ESM.docx]

Additional file 4**.** the Joanna Briggs Institute Checklist for Case Control Studies quality assessment table and descriptions

|  | 1 | 2 | 3 | 4 | 5 | 6 | 7 | 8 | 9 | 10 |
| --- | --- | --- | --- | --- | --- | --- | --- | --- | --- | --- |
| Armstrong et al (2015) | Y | N | Y | N/A | N/A | Y | Y | Y | N/A | Y |
| Armstrong et al (2017) | Y | N | Y | N/A | N/A | Y | Y | Y | N/A | Y |
| Casado et al (2005) | U | N | Y | N/A | N/A | Y | Y | Y | N/A | N |
| Cleare et al (2000) | Y | Y | Y | N/A | N/A | Y | Y | Y | N/A | Y |
| Cleare et al (2001a) | U | Y | Y | N/A | N/A | Y | Y | Y | N/A | N |
| Cleare et al (2001b) | Y | Y | Y | N/A | N/A | Y | Y | Y | N/A | N |
| Hannestad et al (2007) | Y | Y | N | N/A | N/A | Y | Y | Y | N/A | N |
| Inder et al (2005) | N | N | Y | N/A | N/A | Y | Y | Y | N/A | Y |
| Jerjes et al (2006a) | Y | Y | Y | N/A | N/A | Y | Y | Y | N/A | Y |
| Jerjes et al (2006b) | Y | Y | Y | N/A | N/A | Y | Y | Y | N/A | N |
| Jerjes et al (2007) | Y | Y | Y | N/A | N/A | Y | Y | Y | N/A | Y |
| Jones et al (2005a) | Y | N | N | N/A | N/A | Y | Y | Y | N/A | Y |
| Jones et al (2005b) | Y | N | N | N/A | N/A | Y | Y | Y | N/A | Y |
| Lidbury et al (2019) | U | U | N | N/A | N/A | Y | Y | Y | N/A | N |
| Maes et al (2009) | N | N | Y | N/A | N/A | Y | Y | Y | N/A | Y |
| Maloney et al (2006) | N | Y | N | N/A | N/A | Y | Y | Y | N/A | N |
| McGregor et al (2016) | Y | N | Y | N/A | N/A | Y | U | Y | N/A | Y |
| McGregor et al (2019) | Y | N | Y | N/A | N/A | Y | N | Y | N/A | Y |
| Ruiz-Núñez et al (2018) | Y | U | Y | N/A | N/A | Y | Y | Y | N/A | N |
| Scott et al (1998) | U | N | Y | N/A | N/A | Y | Y | Y | N/A | N |
| Young et al (1998) | Y | Y | Y | N/A | N/A | Y | Y | Y | N/A | N |

Items answered as not applicable were removed from the final percentage. Abbreviations: JBI, Joanna Briggs Institute; Y, Yes; N, No; N/A, not applicable; U, unclear.

JBI Checklist items:
1. Were the groups comparable other than the presence of disease in cases or the absence of disease in controls?
2. Were cases and controls matched appropriately?
3. Were the same criteria used for identification of cases and controls?
4. Was exposure measured in a standard, valid and reliable way?
5. Was exposure measured in the same way for cases and controls?
6. Were confounding factors identified?
7. Were strategies to deal with confounding factors stated?
8. Were outcomes assessed in a standard, valid and reliable way for cases and controls?
9. Was the exposure period of interest long enough to be meaningful?
10. Was appropriate statistical analysis used?

**Armstrong et al, 2015**

1. Yes, ME/CFS patients and HC were age- and sex- matched.
2. No, No source information was provided.
3. Yes, ME/CFS patients were diagnosed according to the CCC criteria. HC did not have ME/CFS or any other illness.
4. N/A. There was no exposure – study investigated urinary metabolites in ME/CFS compared to HC.
5. N/A. No exposure.
6. Yes. the following confounding variables were identified: age, sex, medications.
7. Yes. Age and sex- variables were controlled for by exclusion of male participants and those over 60.
8. Yes. 1D 1H Nuclear magnetic resonance spectroscopy was used to measure differences in urinary metabolites.
9. N/A. No exposure.
10. Yes, normality tests were conducted and appropriate statistical tests were selected accordingly (parametric t-tests and Pearson correlations). Data that was not normal were log-converted . Where multivariate analysis was conducted adjustments using Benjamini-Hochberg were made to adjust for multiple comparisons.

**Armstrong et al, 2017**

1. Yes, ME/CFS patients and HC were age- and sex- matched.
2. No, No source information was provided.
3. Yes, ME/CFS patients were diagnosed according to the CCC criteria. HC did not have ME/CFS or any other illness.
4. N/A. There was no exposure – study investigated urinary metabolites in ME/CFS compared to HC.
5. N/A. No exposure.
6. Yes. the following confounding variables were identified: age, sex, medications.
7. Yes. Age and sex- variables were controlled for by exclusion of male participants and those over 60.
8. Yes. 1D 1H Nuclear magnetic resonance spectroscopy was used to measure differences in urinary metabolites.
9. N/A. No exposure.
10. Yes, normality tests were conducted and appropriate statistical tests were selected accordingly (parametric t-tests and Pearson correlations). Data that was not normal were log-converted . Where multivariate analysis was conducted adjustments using Benjamini-Hochberg were made to adjust for multiple comparisons.

**Casado et al, 2004**

1. Unclear. Publication stated that age and gender distributions were as comparable as possible but there was no justification or data provided to indicate whether there were no significant differences between patients and HC.
2. No. No source information was provided.
3. Yes, ME/CFS patients were diagnosed according to Fukuda criteria. Exclusion criteria for all participants include severe physical impairment and medical conditions with symptoms similar to ME/CFS (ie: morbid obesity, autoimmune or inflammatory conditions), severe psychiatric conditions any conditions that effect the HPA axis or autonomic function.
4. N/A. No exposure this study investigates urinary electrophoretic profiles from ME/CFS patients and HC.
5. N/A. No exposure.
6. Yes, confounding variables identified include caffeine intake and medications or factors that effect the HPA axis.
7. Yes, confounding variables were mostly controlled via exclusion, restriction (caffeine) or having a wash-out period of medications.
8. Yes, Capillary electrophoresis was used.
9. N/A. No exposure.
10. No. Normality was considered and appropriate non-parametric tests were conducted including T-tests. ANOVA was conducted for nPh but no adjustments for multiple comparisons were made.

**Cleare et al, 2000**

1. yes, participants were age, sex and BMI matched.
2. Yes, ME/CFS patients attended a clinic at King’s College Hospital in London. HC were staff members or volunteers from King’s college.
3. Yes, ME/CFS patients were defined according to the Fukuda criteria. HC did not have a history of substantial medical problem, ME/CFS or a major psychiatric disorder.
4. N/A. The exposure is not relevant to this systematic review – only baseline data is being reviewed.
5. N/A. See Question 4.
6. Yes, confounding factors were identified including use of medication, a psychiatric condition and menstrual cycle.
7. Yes, those who took medication had a wash out period for at least two months (except contraception). Those with a psychiatric condition were excluded. Patients were scheduled according to the state of their menstrual cycle.
8. Yes, a urine pathology test was conducted to measure growth hormone.
9. N/A. See Question 4.
10. Yes. Normality was considered and appropriate statistical tests were used on data including a t- test for parametric data and mann-whitney U test for non-parametric data.

**Cleare et al, 2001a** Urinary free cortisol

1. Unclear, results stated that gender and weight were well matched, but ME/CFS patients were slightly older. Data showing demographic information was unclear and could not determine whether there were significant differences between age or sex.
2. Yes, ME/CFS patients attended a clinic at King’s College Hospital in London. HC were staff members or volunteers from King’s college.
3. Yes, ME/CFS patients were defined according to the Fukuda criteria. HC did not have a history of substantial medical problems. ME/CFS or a major psychiatric disorder.
4. N/A. There was no exposure. This study investigated urinary free cortisol in ME/CFS patients compared to HC.
5. N/A. No exposure.
6. Yes, confounding factors were identified including use of medication, substance abuse and comorbid conditions.
7. Yes, confounding factors were mitigated through excluding patients that had alcohol or substance abuse either during or two years prior to ME/CFS diagnosis. Those who took medication or had comorbid conditions were placed in an alternative subgroup for comparison and consideration as a cofactor.
8. Yes, technicon Immuno-1 assay of urinary free cortisol measurement was taken.
9. N/A. No exposure.
10. No. No mention of normality tests in the study therefore unclear whether tests selected were appropriate for data distribution. For one- way ANOVA a post hoc test was conducted.

**Cleare et al, 2001b** Hypothalamo

1. yes, participants were age- matched. There were no significant differences in sex between the two groups.
2. Yes, ME/CFS patients attended a clinic at King’s College Hospital in London. HC were staff members or volunteers from King’s college.
3. Yes, ME/CFS patients were defined according to the Fukuda criteria. HC did not have a history of substantial medical problem or a major psychiatric disorder.
4. N/A. The exposure is not relevant to this systematic review – only baseline data is being reviewed.
5. N/A. See Question 4.
6. Yes, confounding factors were identified including use of medication, a psychiatric condition and menstrual cycle.
7. Yes, those who took medication had a wash out period for at least two months (except contraception). Those with a psychiatric condition were excluded. Patients were scheduled according to the state of their menstrual cycle.
8. Yes, a human Cortisol-releasing hormone blood test was conducted.
9. N/A. See Question 4.
10. No. No mention of normality tests in the study therefore unclear whether tests selected were appropriate for data distribution. No indication of a post-hoc test being conducted for one- way ANOVA.

**Hannestad et al, 2006**

1. Yes, ME/CFS patients and HC were age- and sex- matched
2. Yes, ME/CFS patients were from the outpatient clinic at Karolinska University Hospital and HC were staff members from the same hospital.
3. No, ME/CFS patients met the Fukuda criteria, however, no criteria was provided for the recruitment of HC.
4. N/A. No exposure this study investigated excretion levels of β-Alanine and γ-aminobutyric acid
5. N/A. No exposure.
6. Yes, confounding factors such as complete urine samples and urine creatinine levels were considered
7. Yes, those with incomplete urine collections and small quantities of creatinine excretion were excluded from the study.
8. Yes, this study assessed levels of β-Alanine and γ-aminobutyric acid using isotype gas chromatography mass spectrometry.
9. N/A. No exposure.
10. No. No mention of justification of selection of statistical tests. Correction of skewness was made.

**Inder et al, 2005**

1. No, participants were age- matched, however, no information was provided on the sex of participants.
2. No, no source information was provided.
3. Yes, ME/CFS patients were defined according to the Fukuda criteria. HC did not meet criteria for ME/CFS and had normal pathology test results.
4. N/A. The exposure is not relevant to this systematic review – only baseline data is being reviewed.
5. N/A. See Question 4.
6. Yes, confounding factors include drug abuse, eating disorder, major depressive illness or were taking medication known to affect the HPA axis such as corticosteroids
7. Yes, confounding variables were mitigated through exclusion.
8. Yes, cortisol was measured using an enzyme-linked immunosorbent assay (ELISA). ACTH was measured by immunoradiometric assay and IGF-1 was measured via radioimmunoassay.
9. N/A. See Question 4.
10. Yes. Normality was considered. An unpaired t-test was selected when data followed a normal distribution or a mann-whitney U test was selected if the data was not normally distributed to assess differences in basal levels of 24 hour urinary free cortisol, DHEAs and IGF-1. A post- hoc analysis was conducted.

**Jerjes et al, 2006a (diurinal excretion)**

1. Yes, there were no significant differences in age, and BMI. Participants were matched for sex.
2. Yes, ME/CFS patients were recruited from King’s college Hospital. HC were either staff or students from King’s College Hospital.
3. ME/CFS patients met the Fukuda criteria. HC were assessed by a nurse to be in good health without any serious medical illness or history of psychiatric illness.
4. N/A. No exposure. This study investigates diurnal excretion of urinary cortisol, cortisone, and cortisol metabolites in ME/CFS patients compared to HC.
5. N/A. No exposure.
6. Yes, confounding variables that were identified include medications that effect the HPA axis. Alcohol and caffeine and eating and sleep habits were also considered potential confounding variables
7. Yes, confounding medications were mitigated via exclusion. Alcohol and caffeine intake was limited but not avoided fully as changes in habit may also affect HPA axis. Participants has a habitual bedtime routine.
8. Yes, radioimmunoassay and high-resolution gas chromatography of methyloxime-trimethylsilyl ether (MO-TMS)
9. N/A. No exposure.
10. Yes, normality was conducted and an appropriate statistical test was chosen based on outcome. ANOVA was also conducted to determine differences in cortisol levels throughout the day.

**Jerjes et al, 2006b (urinary cortisol)**

1. Yes, there were no significant differences in age, sex, and BMI.
2. Yes, ME/CFS patients were recruited from King’s college Hospital. HC were either staff or students from King’s College Hospital.
3. Yes, ME/CFS patients met the Fukuda criteria. HC were assessed by a nurse to be in good health without any serious medical illness or history of psychiatric illness.
4. N/A. No exposure. This study investigates urinary cortisol and cortisol metabolite excredition in ME/CFS patients compared to HC.
5. N/A. No exposure.
6. Yes, confounding variables that were identified include medications that effect the HPA axis, having normal dietary and sleep habits.
7. Yes, confounding variables were mitigated via exclusion.
8. Yes, radioimmunoassay and high-resolution gas chromatography of methyloxime-trimethylsilyl ether (MO-TMS)
9. N/A. No exposure.
10. No, normality was conducted and an appropriate statistical test was chosen based on outcome; however a general linear regression method was conducted and there were no corrections for multiple comparisons.

**Jerjes et al, 2007 (Enhanced feedback)**

1. Yes, there were no significant differences in age, and BMI. Participants were matched for sex.
2. Yes, ME/CFS patients were recruited from King’s college Hospital. HC were either staff or students from King’s College Hospital.
3. Yes, ME/CFS patients met the Fukuda criteria. HC were assessed by a nurse to be in good health without any serious medical illness or history of psychiatric illness.
4. N/A. No exposure. This study investigates urine cortisol metabolites in ME/CFS patients compared to HC.
5. N/A. No exposure.
6. Yes, confounding variables that were identified include medications that effect the HPA axis. Alcohol and caffeine and eating and sleep habits were also considered potential confounding variables
7. Yes, confounding medications were mitigated via exclusion. Alcohol and caffeine intake was limited but not avoided fully as changes in habit may also affect HPA axis. Participants has a habitual bedtime routine.
8. Yes, radioimmunoassay and high-resolution gas chromatography of methyloxime-trimethylsilyl ether (MO-TMS) was used.
9. N/A. No exposure.
10. Yes, data was assessed for normality. Abnormally distributed data was log converted. T test was utilised for normally distributed data.

**Jones et al, 2005a (plasma and urinary)**

1. Yes, participants were age and sex- matched
2. No, no demographic information provided.
3. No, Fukuda criteria was used for ME/CFS patients, however, the healthy controls were not well defined.
4. N/A, no exposure. This study investigated urinary carnitine and acylcarnitine levels in ME/CFS compared to HC.
5. N/A. No exposure.
6. Yes, confounding variables that were identified include medication, the presence of a psychiatric condition or, those who smoked.
7. Yes, confounding variables were mitigated via exclusion.
8. Yes, hydrophilic interaction liquid chromatography was used.
9. N/A. No exposure.
10. Yes, unpaired independent t-test and Mann-Whitney U test was conducted on the basis of data distribution. (did not mention normality but implied through methodology)

**Jones et al, 2005b (urinary and plasma)**

1. Yes, participants were age and sex- matched
2. No, no demographic information provided.
3. No, Fukuda criteria was used for ME/CFS patients, however, the healthy controls were not well defined.
4. N/A, no exposure. This study investigated urinary organic and amino acids in ME/CFS compared to HC.
5. N/A. No exposure.
6. Yes, confounding variables that were identified include medication, the presence of a psychiatric condition or, those who smoked.
7. Yes, confounding variables were mitigated via exclusion.
8. Yes, hydrophilic interaction liquid chromatography was used.
9. N/A. No exposure.
10. Yes, distribution was assessed for normality and an unpaired independent t-test and Mann-Whitney U test was conducted on the basis of data distribution.

**Lidbury et al, 2019**

1. Unclear, age matched but not unclear if sex- matched.
2. Unclear, ME/CFS patients were recruited from “CFS Discovery” located in Donvale, Victoria (Australia). Control recruitment location was unclear.
3. No, the ICC was used to diagnose patients. Criteria was not described for HC.
4. N/A. No exposure.
5. N/A. See 4.
6. Yes, comorbidities were identified as a potential confounding variable.
7. Yes, confounding variables were mitigated via exclusion.
8. Yes, 24-hour excretion was measured using standardised pathology tests.
9. N/A. See 4.
10. No, normality was assessed using the Kolmogorov- Smirnov test. A mann-whitney U and Kruskal- wallis non- parametric test was selected based on the outcome of the normality test. Unclear if adjustments for multiple comparisons for Kruskal-wallis were made.

**Maes et al, 2009**

1. No, no mention of age- and sex- matching.
2. No, all patients were admitted to the Maes clinics, Antwerp, Belgium. No demographic information was supplied for the HC.
3. Yes, ME/CFS patients were defined according to the Fukuda criteria. HC were excluded if they have medical illnesses such as inflammatory bowel disorders, diabetes type 1 or type 2, hypertension or atherosclerosis.
4. N/A. No exposure. This study investigates 8-hydroxy-deoxyguanosine levels in ME/CFS compared to HC.
5. N/A. No exposure.
6. Yes, confounding factors include participants with a life-time diagnosis of psychiatric DSM IV-R disorders.
7. Yes, confounding factors were mitigated via exclusion.
8. Yes, 8-OHdG was measured using ELISA.
9. N/A. No exposure.
10. Yes, data was normalised using a box-cox transformation. Non- parametric ANOVAs and ANCOVAS were used, and a Dunn test was conducted to determine post-hoc differences.

**Maloney et al, 2006**

1. No, participants were matched on the basis of: age, sex, race, and body mass index, however, following exclusion based on the presence of major depressive disorder or melancholic features the ME/CFS patients and controls were not matched.
2. Yes, participants were located in Wichita, KS, USA.
3. No, ME/CFS patients were diagnosed according to the Fukuda/ Reeves criteria. Criteria was not sufficient for HC (may be available in supporting publication).
4. N/A. No exposure this study investigated allostatic load index in ME/CFS patients compared to HC (cortisol was measured using 24 hour urine sample)
5. N/A. No exposure.
6. Yes, major depressive disorder was considered a confounding variable.
7. Yes, confounding variables were mitigated via exclusion.
8. Yes, cortisol was measured from a 24-hour urine sample using standard clinical protocol.
9. N/A. No exposure.
10. No, logistic regression was used and no mention of normality tests conducted as well as adjustments made for multiple comparisons.

**McGregor et al, 2016**

1. Yes, participants were age- and sex- matched.
2. No, no demographic information was provided.
3. Yes, ME/CFS patients met CCC definition. HC were fatigue free.
4. N/A. No exposure. This study investigated urine metabolomes in relation to renal function in ME/CFS patients compared to HC.
5. N/A. No exposure.
6. Yes, confounding factors identified include medication and oral supplements as well as participants living or related to each other.
7. Unclear, it was not stated how these confounding factors were controlled for in this study.
8. Yes, standard serum biochemistry test on urine was conducted.
9. N/A. no exposure.
10. Yes, data was assessed for normality. Those that do not comply with normality standards were log converted. Parametric tests were used including Pearson correlation coefficients, ANOVA and multivariate analysis. Multiplicity corrections were conducted.

**McGregor et al, 2019**

1. Yes, participants were age- and sex- matched.
2. No, no demographic information was provided.
3. Yes, ME/CFS patients met CCC definition. HC were fatigue free.
4. N/A. No exposure. This study investigated urine metabolomes in relation to renal function in ME/CFS patients compared to HC.
5. N/A. No exposure.
6. Yes, confounding factors identified include medication and oral supplements
7. No, no mention of how authors mitigated confounding factors
8. Yes, standard serum biochemistry test on urine was conducted.
9. N/A. no exposure.
10. Yes, data was assessed for normality. Those that do not comply with normality standards were log converted. Parametric tests were used including Pearson correlation coefficients, ANOVA and multivariate analysis. Multiplicity corrections were conducted.

**Ruiz-Nunez et al, 2018**

1. Yes, ME/CFS patients were age- and sex- matched.
2. Unclear, ME/CFS patients were recruited in the Parkstad clinic in Amsterdam, The Netherlands, however, three participants that didn’t attend this clinic were included and their recruitment location was not stated. HC were recruited in the city of Groningen, the Netherlands.
3. Yes, ME/CFS patients were diagnosed according to Fukuda/ Reeves criteria. Exclusion criteria for HC was the use of any chronic medication, menstruation during urine collection, obesity and hypothyroidism.
4. N/A. No exposure. This study investigated thyroid function in ME/CFS patients compared to HC – in particular 24-hr urinary iodine levels.
5. N/A. No exposure.
6. Yes, confounding factors include: medications that may affect thyroid function or hypothyroidism, pregnancy, and menstruation during urine collection, CRP levels (low-grade inflammation).
7. Yes, confounding variables were mitigated via either exclusion, appropriate scheduling of urine collection or formation of subgroups during analysis.
8. Yes, standardised biochemical measures were used.
9. N/A. No exposure.
10. Yes, normality was considered, and non-parametric tests were selected. Mann-whitney U test and chi squared analysis were conducted (no further adjustment for multiple comparisons are needed for chi square test).

**Scott et al, 1998**

1. Unclear, Unclear if age and sex- matched. A correlation analysis was conducted to assess the affect of age or sex on the sample population but it is unknown whether age- and sex- were appropriately controlled for.
2. No, no source information was supplied.
3. Yes, ME/CFS patients were diagnosed according to Fukuda criteria. HC had a past or current history of chronic fatigue, psychiatric illness, other neurological, endocrine, cardiovascular, renal or hepatic illness.
4. N/A. No exposure. This study investigates urinary cortisol levels in ME/CFS patients compared to HC.
5. N/A. No exposure.
6. Yes, Confounding factors include: history of heavy alcohol consumption or illicit drug use or any medications known to affect the HPA axis (four weeks prior to testing).
7. Yes, Confounding factors were mitigated either by exclusion or by a wash out period for interacting medications.
8. Yes, cortisol levels were measured using radioimmunoassay.
9. N/A. No exposure.
10. No, no mention of normality to determine appropriateness of test selected were provided. No adjustments were made for ANOVA or correlation analysis.

**Young et al, 1998**

1. Yes, ME/CFS patients and HC were matched for age, gender and weight.
2. Yes, ME/CFS patients were recruited from a hospital infectious disease clinic. HC were hospital employees.
3. Yes, ME/CFS patients were diagnosed according to the Fukuda criteria. HC did not have any current physical or psychiatric disorder.
4. N/A. No exposure. This study investigated urinary cortisol in ME/CFS patients compared to HC.
5. N/A. No exposure.
6. Yes. Potential confounding factors identified include medication.
7. Yes. Confounding factors were mitigated.
8. Yes, Urinary-free cortisol was measured using a standard extraction radioimmunoassay kit
9. N/A. No exposure.
10. No. Unpaired T -tests were used to analyse area under the curve data. Unclear about the data distribution and therefore appropriateness of the test selected.
